# Attitudes, beliefs, and perceptions of caregivers and rehabilitation providers about disabled children’s sleep health: a qualitative study

**DOI:** 10.1186/1471-2431-14-245

**Published:** 2014-10-01

**Authors:** Xiaoli Chen, Bizu Gelaye, Juan Carlos Velez, Micah Pepper, Sara Gorman, Clarita Barbosa, Ross D Zafonte, Susan Redline, Michelle A Williams

**Affiliations:** Department of Epidemiology, Harvard School of Public Health, Boston, MA 02115 USA; Centro de Rehabilitación Club de Leones Cruz del Sur, Punta Arenas, Chile; Department of Health Policy & Management, Columbia University Mailman School of Public, Health, New York, USA; Spaulding Rehabilitation Hospital, Massachusetts General Hospital Physical Medicine and Rehabilitation Service, Boston, MA 02114 USA; Division of Sleep and Circadian Disorders, Brigham and Women’s Hospital, Boston, MA 02115 USA; Department of Medicine, Harvard Medical School, Boston, MA 02115 USA

**Keywords:** Child, Disability, Sleep hygiene, Parent, Health care provider, Focus group

## Abstract

**Background:**

Children with disabilities are more likely to have sleep disturbances than children without disabilities. Identifying attitudes, beliefs, knowledge, and perceptions of caregivers and health professionals is essential in developing effective intervention programs to improve disabled children’s sleep health. However, no such qualitative data about adults who have key roles in the life and daytime activities of children with disabilities are available. This qualitative study aimed to understand attitudes, beliefs, knowledge, and perceptions about disabled children’s sleep hygiene among caregivers and rehabilitation providers of children with disabilities.

**Methods:**

Twenty seven adults, including nine primary caregivers and eighteen rehabilitation providers, participated in five focus group discussions between September and December 2012 at the Rehabilitation Center in Punta Arenas, Chile. A trained facilitator guided focus group discussions using a semi-structured script. Audiotapes and transcripts of focus group discussions were reviewed and analyzed for recurrent themes.

**Results:**

Participants identified seven themes related to children’s sleep hygiene: lifestyle behaviors, family factors, children’s disabilities and/or comorbidities, environmental factors, adults’ responsibilities for children’s sleep, perception of good sleep, and parental distress about children’s sleep problems. While both caregivers and rehabilitation providers recognized the importance of sleep for children’s health and functioning, they differed in their understanding of how sleep hygiene practices influence sleep. Rehabilitation providers recognized the negative influence of electronics on sleep and the positive influence of sleep routines. In contrast, caregivers reported use of television/movie watching and stimulants as coping strategies for managing children’s sleep problems.

**Conclusions:**

Caregivers may benefit from better understanding the influence of electronics and stimulant use on child sleep health. Rehabilitation providers are well positioned to provide educational messages to both children and caregivers in order to change their attitudes, perceptions, and practices surrounding sleep. These qualitative data are valuable in developing intervention programs aimed at improving sleep health among children with disabilities.

## Background

At least 93 million children are living with disabilities worldwide [1]. Children with disabilities such as attention-deficit and/or hyperactivity disorder (ADHD) are more likely to have sleep disturbances than children without disabilities [2–9], and may warrant particular attention for health promotion and disease prevention. Identifying attitudes, beliefs, knowledge, and perceptions of caregivers and health professionals who treat children with disabilities is essential in developing effective intervention programs to improve children’s sleep health. To our knowledge, no such qualitative data about adults who have key roles in the life and daytime activities of children with disabilities are available. The lack of such qualitative data about children’s sleep among caregivers and health professionals may contribute to the persistent high prevalence of sleep disturbances in disabled children. Since successful interventions should reflect the views of targeted populations [10], focus groups are commonly used because they can efficiently explore the attitudes, beliefs, and perceptions of the participants.

In this qualitative study, we conducted focus group interviews about children’s sleep with primary caregivers and rehabilitation providers of children with disabilities. The aims of this study were to: 1) understand the attitudes, beliefs, knowledge, and perceptions of caregivers and rehabilitation providers of children with disabilities regarding children’s sleep; 2) identify factors that could facilitate or impair children’s sleep hygiene; and 3) identify areas for intervention and improvement of children’s sleep health.

## Methods

### Participants

The Chile Pediatric and Adult Sleep and Stress Study (CPASS) was conducted in the Patagonia Region of Chile. The study was established in September 2012 at the Centro de Rehabilitacion Club de Leones Cruz del Sur in Punta Arenas, Chile. The present qualitative study was conducted between September and December 2012 at the center.

Using a recruitment script, a research staff member approached primary caregivers of children with disabilities when caregivers checked in for their children’s appointment. Children with disabilities were those who used assistive devices and/or received routine clinical care at the center for their chronic disorders such as speech and/or motor delay, ADHD, or other types of mental disorders including Down syndrome and autism. A recruitment flyer was posted in the staff lounge asking rehabilitation providers to contact the research staff if they were interested in participating in the study. Nine adult caregivers aged ≥18 years who spoke and read Spanish and who were without intellectual disabilities participated in 2 focus group discussions (4–5 participants per group). Three additional focus groups were conducted among 18 rehabilitation providers who cared for children with disabilities, including physicians, physical/speech therapists, psychologists, and special education teachers (6 participants per group).

This study was approved by the institutional review boards of Centro de Rehabilitacion Club de Leones Cruz del Sur (IRB # 016) in Punta Arenas, Chile and Harvard School of Public Health (IRB # 22797–101) in Boston, USA. All participants provided written informed consent.

### Focus groups

All focus group discussion sessions were held in a meeting room at the center. Before the start of discussions, participants completed a brief survey providing information on sociodemographics, sleep medicine training background (for rehabilitation providers only), and the number of years that caregivers had been taking care of, or rehabilitation providers had been working with, children with disabilities. At the beginning of each session, a trained facilitator who was a psychologist at the center explained the purpose of the study, and then conducted semi-structured sessions using a discussion guide developed by the research team. Of note, the facilitator was not a member of the research team. A trained note-taker took the discussion notes. For each idea discussed, general open-ended questions were followed by probing questions.

### Data analysis

All focus group discussions were digitally audio-recorded and transcribed verbatim by a professional transcriber. Audio recordings were translated, transcribed, and annotated to clarify linguistic and cultural information. One research team member who was bilingual in English and Spanish assured the accuracy of the translation of the transcripts. The process of triangulation was used to read and code the transcripts to enhance the quality and credibility of qualitative analyses [11]. Two researchers coded the transcripts independently using thematic codes consistent with original aims of the study. The method of repetition was used to identify themes, while cutting and sorting processes were applied to code the transcripts [12]. Disagreements about the meanings of participants’ comments were resolved through discussion and consensus. The final coding schemes were applied to all transcripts. To establish inter-rater reliability between the transcript coders, intra-class correlation coefficient (ICC) was calculated based on the method of Shrout and Fleiss [13]. Thematic analyses were conducted of participants’ comments and discussions about their attitudes, beliefs, knowledge, and perceptions of children’s sleep. To protect the identify of participants, pseudonyms are used.

## Results

### Participants’ characteristics

The average age of focus group participants was 43.0 (range: 25.2-64.2) years for caregivers and 35.4 (range: 24.2-49.3) years for rehabilitation providers, respectively. All caregivers were women (8 mothers and 1 grandmother), 1 had a middle school degree, 5 had high school degrees, and 3 had college degrees (Table 1). Five caregivers were housewives, 3 had full-time jobs, and 1 caregiver was unemployed. Caregivers reported having taken care of children for an average of 8.5 (range: 6–13) years. Rehabilitation providers reported having worked with disabled children for an average of 6.3 (range: 1–12) years. Eight out of 18 rehabilitation providers reported professional experience with sleep medicine or sleep health hygiene; only 2 had sleep medicine training.

**Table 1:** **Characteristics of 27 focus group participants**

Focus group	Number of participants	Mean age (range), years	Gender	Education status	Employment status
**Group 1**	4 caregivers	39.7 (25.2-48.4)	4 women	1 had a middle school degree, 1 had a high school degree, 2 had college degrees	2 were housewives, 2 had full-time jobs
**Group 2**	5 caregivers	46.1 (34.5-64.2)	5 women	4 had high school degrees, 1 had a college degree	3 were housewives, 1 was unemployed, 1 had a full-time job
**Group 3**	6 rehabilitation providers	32.5 (24.3-46.9)	3 men, 3 women	6 had college degrees	5 had full-time jobs, 1 had a part-time job
**Group 4**	6 rehabilitation providers	36.5 (26.8-43.4)	3 men, 3 women	6 had college degrees	6 had full-time jobs
**Group 5**	6 rehabilitation providers	34.9 (24.2-49.3)	2 men, 4 women	3 had technical school degrees, 3 had college degrees	5 had a full-time job, 1 had a part-time job

### Major themes

In this study, the inter-rater ICC value was 0.90, indicating excellent inter-rater reliability between the two transcript coders. Seven themes were identified as risk factors for children’s sleep (Table 2; Table 3 for representative quotations): 1) lifestyle factors; 2) family factors; 3) children’s disability/disease-related factors; 4) environmental factors; 5) adults’ responsibilities for children’s sleep; 6) perceptions of good sleep; 7) parental distress about children’s sleep problems.

**Table 2:** **Focus group participants’ attitudes, beliefs, and perceptions about factors related to children’s sleep**

Theme	Code	Total quotes (total: 27) N (%)	Caregivers (total: 9) N (%)	Rehabilitation providers (total: 18) N (%)
**Lifestyle factors**	Routine/rule/individual preference	16 (59.3%)	5 (55.6%)	11 (61.1%)
	Physical activity	8 (29.6%)	2 (22.2%)	6 (33.3%)
	Screen time	4 (14.8%)	1 (11.1%)	3 (16.7%)
	Diet/hunger	4 (14.8%)	1 (11.1%)	3 (16.7%)
	Stimulant use	3 (11.1%)	0 (0.0%)	3 (16.7%)
**Family factors**	Family routine	16 (59.3%)	5 (55.6%)	11 (61.1%)
	Parents’ work schedule	7 (25.9%)	3 (33.3%)	4 (22.2%)
	Adapting to child/coping	3 (11.1%)	0 (0.0%)	3 (16.7%)
**Children’s disability/disease-related factors**	Diagnosis/comorbidity	14 (51.9%)	0 (0.0%)	14 (77.8%)
	Medication use	13 (48.1%)	1 (11.1%)	12 (66.7%)
	Sleep problems	9 (33.3%)	8 (88.9%)	1 (5.6%)
**Environmental factors**	Light/sunlight	6 (22.2%)	1 (11.1%)	5 (27.8%)
	Noise	5 (18.5%)	1 (11.1%)	4 (22.2%)
	Quality of sleeping space	3 (11.1%)	1 (11.1%)	2 (11.1%)
**Adults’ responsibility for children’s sleep**	Parents’ responsibility	4 (14.8%)	1 (11.1%)	3 (16.7%)
	Family’s responsibility	2 (7.4%)	0 (0.0%)	2 (11.1%)
	Physicians’ responsibility	3 (11.1%)	2 (22.2%)	1 (5.6%)
**Perceptions of good sleep**	Feeling next day	14 (51.9%)	5 (55.6%)	9 (50.0%)
	Interrupted sleep or not	9 (33.3%)	3 (33.3%)	6 (33.3%)
	Quality of sleep	6 (22.2%)	2 (22.2%)	4 (22.2%)
	Quantity of sleep	8 (29.6%)	4 (44.4%)	4 (22.2%)
	Ideal sleep duration for children aged			
	<5 years old was ≥10 hours	25 (92.6%)	8 (88.9%)	17 (94.4%)
	6-12 years old: 9–10 hours	20 (74.1%)	6 (66.7%)	14 (77.8%)
	13-18 years old: 8 hours	14 (51.8%)	5 (55.5%)	9 (50.0%)
	Waking up on one’s own	4 (14.8%)	2 (22.2%)	2 (11.1%)
	Bedtime	2 (7.4%)	2 (22.2%)	0 (0.0%)
**Parental distress**	Anxiety	6 (22.2%)	4 (44.4%)	2 (11.1%)
	Frustration	3 (11.1%)	3 (33.3%)	0 (0.0%)

**Table 3:** **Themes, codes, and representative quotes from 27 focus group participants**

Theme	Code	Participants’ sociodemographic characteristics	Representative quotes
**Lifestyle factors**	Routine/rule, and individual preference	Mother, housewife, Middle school degree	“I think they get used to their school schedule, because Diego, even though it is a weekend he will be up at 8.”
	Physical activity	Grandmother, housewife, high school degree	“When my grandson was a year and a half old he didn’t sleep at night nor during the day, we put him to bed at 9:00 and he would fall asleep at 10:00 but he wouldn’t fall asleep so that’s when the doctor started him on melatonin, because a child that doesn’t walk and spends most of the day on the floor has very little activity, he doesn’t get tired, we took him out for walks but it was the same, we would get home and he would sleep for an hour, we were going crazy.”
	Screen time	Physician, medical college degree	“I think that school children are not sleeping enough, well, parents do not have the same control as before, for example, they stay on the Internet, on Facebook at night and I have seen with my own children that their friends are connected at 3 in the morning, I mean there are people who stay on line all night and they are not sleeping.”
	Diet	Mother, engineer, college degree	“But actually what wakes us all up is our appetite, I mean, one of the kids gets hungry and right ways they are downstairs drinking juice or looking for something to eat.”
	Stimulant use	Physician, medical college degree	“…it is very common now to see those popular coffee machines all over the place, and one sees school kids and adolescents in their first stages of adolescence freely consuming this, and one knows that this has an effect on sleeping, where the sleep is maintained and should be a way of resting and one sees them drinking coffee.”
**Family factors**	Family routine	Mother, public employee, college degree	“…things that influence the issue of sleep for my son is family organization… in my particular case this structure is a bit broken, because as of 5 months his father is working in another place.”
	Parents’ work schedule	Physician, medical college degree	“Yes, because sometimes the ones who work on shifts for example, one sees that the routine of the child is different on the days when the father is not home than on the 7 days when the father is home, right? On the week where the father is not home the schedules are met by the person in charge, in this case the mother who does not work on shifts…”
	Adapting to child/coping	Physical therapist, college degree	“What happens in society is that there is a tendency for parents to adapt to the child when they have special needs, a routine is made, and if they have to go to bed they have to go to bed, if they have to wake up early they must wake up early.”
**Children’s disability/disease-related factors**	Diagnosis/comorbidity	Psychologist, college degree	“…in the kids with attention disorders, who are hyperactive, who undergo treatments that make them very active during the day, but at night it’s like they get unplugged and fall dead asleep and do not wake up, I would think that in that particular sleep it is a very rested one and sufficiently long.”
	Medication use	Speech therapist, college degree	“…if the medications influence their dreams, it is possible that their sleep will be interrupted and will need more hours in order to rest.”
	Sleep problems	Mother, engineer, college degree	“My kids have nightmares, they talk and wake up scared, there aren’t any other sounds they only speak once in a while, they sit up but that’s it, sometimes they wake up crying, the one in the middle is always more scared.”
**Environmental factors**	Light	Special education teacher, college degree	“I think that there are many factors, a set of things, the environment at home may be one, the rules at home. For example, now those of us in Magellan have more light, at 10:00 pm it’s still clear. This may affect a child’s sleep routine.”
	Noise	Mother, unemployed, high school degree	“Well in my case, if there is any sort of noise she wakes up.”
	Quality of sleeping space	Mother, housewife, high school degree	“The environment is fundamental and the other thing I think the child should have space, comfort, tranquility and for example the main thing is a good bed, the proper mattress, because if the child has a bad bed he won’t sleep properly.”
		Special education teacher, college degree	“A warm room, a comfortable bed and comfortable sheets, and no worries because when you have worries it doesn’t allow you to sleep well”
**Responsibility**	Parents’ responsibility	Physical therapist, college degree	“One can give a wide range of possibilities, but the parents must choose.”
	Family’s responsibility	Physician, medical college degree	“Yes, it literally is a frequent question when you are a doctor and the reality of the child will show, and of the 95% that do not have a problem or a cause, arrange to have epilepsy or another disorder. The problem is in inadequate acts, and until the family understands that it is because of their actions, it is difficult to make changes, but to complain and not accept it even though we try to show them different way of what is not functioning well even though they are suffering because there are families that have almost separated because usually it is one person that assumes all responsibilities because the other has to rest for work and as the other has to stay home.”
	Physicians’ responsibility	Physician, medical college degree	“[Caregivers] want a magic solution, that we give them something so the child will sleep and they can count on this to make the child go to sleep when she needs the child to go to sleep.”
**Perceptions of good sleep**	Feeling next day	Physical therapist, college degree	“I think bad sleep and good sleep have to do with the feeling that you have when you wake up, you feel that you rested, you feel that it was restful for you, you feel you can start the day well or it is hard to start the day, or you feel you needed more hours.”
	Interrupted sleep	Mother	“That I get woken up a few times during the night.”
	Quality of sleep	Physical therapist, college degree	“A good sleep is a sleep that reaches all stages of restful sleep.”
	Quantity of sleep	Mother, high school degree	“I think good sleep has to do with time and quantity…”
	Ideal sleep duration for children	Rehabilitation provider, college degree	(as for adolescents aged 13–18 years) “yes, 8 hours, it’s like adult sleep.”
	Waking up on one’s own	Rehabilitation provider, technical school degree	“A good sleep is when I wake up without an external factor like an alarm clock or something like that, it doesn’t happen much, but when it does it means that you slept enough, you wake up automatically.”
	Bedtime	Mother	“If I go to bed late, I will be tired during the day and for example if I put them to bed late I think they too will be tired.”
**Parental distress**	Anxiety	Mother, housewife, high school degree	“Well I have to wake up Anna, because she doesn’t wake up by herself…if she has to go to school, and as you say I make her more nervous, because at 9 when I put her to bed I’m nervous because she has to go to bed so I make her hurry, really I pressure her, in the mornings I pressure her to get up, because she doesn’t get up alone.”
	Frustration	Mother, housewife, high school degree	“My husband and I ‘Dave go to bed, Dave go to sleep, 1, 2, 3’ and I go over there and no, he hides, he goes round one thing or another, but tell him stories no, I only scream.”
**Coping strategies**	Staying with child	Mother, public employee, college degree	“I accompany him for a while, if I have something to do I tell him and I leave him alone, but if I don’t, I stay with him.”
	Sleep medication use	Grandmother, housewife, high school degree	“First as a grandmother, I used to cover him with a blanket, and since he didn’t fall asleep the doctor gave him 2 melatonin.”
	Bathing	Mother, housewife, high School degree	“Give him a bath.”
	Story-telling, comforting, and/or affection	Mother, college degree	“Tell them stories, show them affection so they can relax.”
	Watching television/movies	Mother, housewife, high school degree	“I have been noticing that Anna goes to bed and stays with the television on, even if it’s really low, she falls asleep faster than when I turn off the lights and tell her to go to sleep, it takes her around 45 minutes, but with the television on it takes 15 to 20 minutes.”
**Sleep management advice from health providers**	Establish sleeping routine	Special education teacher, college degree	“Yes in the teacher-parent meeting, I mentioned that children were sleepy and I advised parents to create a sleeping routine, so the learning process was effective.”
	Control use of electronics	Physical therapist, college degree	“For example, too much computer use prior to going to bed, too much television, which are obviously stimulating, before bedtime.”
	Control stimulant use	Physical therapist, college degree	“Food also, soft drinks, for example, there are children who cannot sleep, and you can tell he had a liter of coke, then obviously he is going to be really active.”

#### Lifestyle factors

Participants in all focus groups discussed how lifestyle behaviors affected children’s sleep. Five of 9 caregivers (56%) and 11 of 18 rehabilitation providers (61%) stated that children’s routines, family/school schedules or rules, and individual preferences affected children’s sleep patterns.

Participants also discussed the associations between physical activity and sleep. Several participants believed that physical activity was related to children’s sleep, and sleep medicine could help children with sleep problems due to lack of daytime activity. Notably, several participants believed that children’s sleep health was affected by the time children spent watching television, playing video games, and using computers/internet and other electronic devices. One mother commented: “Yes, he wakes up on his own at that time, most of the time I am sleeping at that time and he is sitting on the couch watching TV. It is not hunger or anything else, it is the TV”.

Participants also mentioned the associations between diet and/or hunger and children’s sleep. Approximately 17% of rehabilitation providers noted that modern lifestyle changes such as consumption of coffee and soft drinks were connected with children’s sleep. However, no caregivers mentioned stimulant use when discussing sleep-related risk factors.

#### Family factors

Most participants believed that family routines, parents’ work schedules, and their capacity to accommodate the needs of children with disabilities could affect children’s sleep. Several rehabilitation providers discussed the potential effect of adapting activities and developing routines to accommodate the special needs for improving sleep health among children with disabilities. Overall, participants recognized that various family factors were related to child sleep, highlighting the importance of healthy sleep habits (e.g., establishing a soothing pre-sleep routine) in enhancing children’s sleep health.

#### Children’s disability/disease-related factors

Many rehabilitation providers (78%) stated that diagnosis-specific features and comorbid conditions were related to children’s sleep. Two-thirds of rehabilitation providers also expressed their concern about side effects of medications on child sleep; 1 caregiver mentioned this.

Eight caregivers (89%) reported that their children had sleep problems such as nightmares, teeth grinding, snoring, talking/noises during sleep, and nocturnal awakening. As a mother described: “My kids have nightmares, they talk and wake up scared, there aren’t any other sounds, they only speak once in a while, they sit up but that’s it, sometimes they wake up crying, the one in the middle is always more scared.” Another mother stated: “He sucks his tongue, grinds his teeth and snores, makes noises with his mouth, I wake him up sometimes because I think that he ruins his teeth.” Overall, most caregivers were aware of their children’s sleep problems, and expressed concern about children’s sleep health.

#### Environmental factors

Several participants believed that environmental factors including light, noise, and sleeping space could affect bedtime and sleep quality. Some participants mentioned the summer sunlight, especially in the Magellan Region in Chile, in relation to sleep. Several participants believed that noise was an environmental factor affecting sleep health. In general, participants perceived that sleeping environment quality was important.

#### Adults’ responsibilities for children’s sleep

Some caregivers believed that health professionals should help with their children’s sleep problems. Caregivers expressed a desire to rely on physicians who take responsibility. They believed that health professionals could provide sleep advice and prescribe sleep medications. In contrast, rehabilitation providers believed that parents and families should take responsibility for their children’s sleep hygiene. Rehabilitation providers expressed the opinion that it should be parents’ or families’ responsibilities to create a routine for children and to enforce sleeping rules.

#### Perceptions of good sleep

Most participants believed that good sleep should produce energized feelings the next day and featured: an early bedtime; no interruptions; long sleep; high sleep quality; and waking up on one’s own. Some caregivers perceived the importance of early bedtime and quiet bedtime activities (e.g., book reading). Both caregivers and rehabilitation providers believed that the appropriate amount of sleep was important, depending on children’s ages. Most participants believed that children <5 years of age should sleep at least 10 hours. However, more than two-thirds of caregivers and rehabilitation providers believed that children 6–12 years of age need 9–10 hours of sleep. Fifty percent of caregivers and rehabilitation providers believed that adolescents 13–18 years of age only need 8 hours of sleep.

#### Parental distress about children’s sleep problems

Several caregivers reported adverse emotional responses due to children’s sleep problems, such as anxiety and frustration. A mother described: “My husband and I always say: ‘Dave go to bed, Dave go to sleep, 1, 2, 3’ and I go over these and no, he hides, he goes round one thing or another”.

We also examined caregivers’ coping strategies with children’s sleep problems and sleep management advice from rehabilitation providers (Table 4). Caregivers reported using various methods to help children fall asleep, including staying with children, using sleep medicine, bathing, telling stories, and/or watching television or movies during bedtime. Some caregivers would allow the children to watch television/movies to deal with sleep problems.

**Table 4:** **Caregivers’ coping strategies with children’s sleep problems and rehabilitation providers’ sleep management advice**

Theme	Code	Total quotes (total: 27) N (%)	Caregivers (total: 9) N (%)	Rehabilitation providers (total: 18) N (%)
**Caregivers’ coping strategies**	Staying with child	3 (33.3%)	3 (33.3%)	-
	Using sleep medication	2 (22.2%)	2 (22.2%)	-
	Bathing	2 (22.2%)	2 (22.2%)	-
	Story-telling, comforting, affection	2 (22.2%)	2 (22.2%)	-
	Watching television/movie	2 (22.2%)	2 (22.2%)	-
**Rehabilitation providers’ sleep management advice**	Establish sleeping routine	5 (27.8%)	-	5 (27.8%)
	Control use of electronics	4 (22.2%)	-	4 (22.2%)
	Control stimulant use	1 (5.6%)	-	1 (5.6%)

Rehabilitation providers perceived that the use of electronic devices prior to bedtime was related to poor sleep, and believed that the control of television/internet use and stimulant use and the establishment of sleep routines were essential. As one special education teacher stated: “Yes, in the teacher-parent meeting, I mentioned that children were sleepy and I advised parents to create a sleeping routine, so the learning process was effective.” One participant mentioned the control of stimulant use: “…there are children who cannot sleep, and you can tell he had a liter of coke, then obviously he is going to be really active”.

## Discussion

Focus group participants identified 7 themes related to children’s sleep, such as lifestyle behaviors, family factors, and children’s disabilities or comorbidities. Caregivers reported using various methods to help children fall asleep during bedtime, some of which are inconsistent with good sleep hygiene practices, such as staying with children, watching TV/movies, and using sleep medicine. In contrast, sleep management advice from rehabilitation providers was highly appropriate, and included control of television/internet and stimulant use, and the establishment of sleep routines for children. To our knowledge, this is the first study to examine attitudes, beliefs, knowledge, and perceptions about children’s sleep from both caregivers and rehabilitation providers of children with disabilities. The observations that caregivers and rehabilitation providers had opposing perspectives regarding the influences of lifestyle characteristics such as electronics, coffee consumption, and medication use on child sleep, and that most individuals had high levels of concern over children’s sleep but often under-estimated sleep needs of children may be of fundamental importance for informing the design of intervention programs aimed at improving sleep health among children with disabilities.

Various factors may play important roles in children’s sleep [14]. We found that caregivers exhibited awareness of behavioral, environmental, and family factors. Most caregivers realized that family schedules and routines, as well as sleeping space and noise, were related to children’s sleep. Caregivers and rehabilitation providers believed that sleep duration and sleep quality were important determinants of good sleep. However, many caregivers and health professionals endorsed fewer than the ideal amount of sleep hours for school-aged children, indicating that they may have inappropriate perceptions of sleep needs for children. Our previous study reported that school-aged children need more sleep than the amounts that participants perceived as ideal [15]. Individuals from divergent cultures may have different perceptions and views concerning the nature and importance of sleep. We note that investigators have documented cultural differences in sleep attitudes and beliefs across populations [16, 17].

Rehabilitation providers expressed particular concern about the effects of electronics and stimulant use on children’s sleep. Although caregivers believed that behavioral factors were related to children’s sleep, none of them mentioned stimulant use as a possible influence. This suggests that caregivers may be unaware of the connection between stimulant beverage consumption and poor sleep. Caffeine consumption is becoming common among children and youths globally [18, 19]. Our recent study showed that caffeinated beverages and other stimulant use were significantly associated with poor sleep among college students in Chile [18]. Increasing evidence has also shown that television viewing and computer use are related to too little sleep for children [20, 21]. The National Sleep Foundation Sleep in America Poll reported that caffeine intake and the use of new technology are associated with shortened sleep among US children [21]. Our study underscores the need to educate children and caregivers about the influence of caffeine consumption and screen time on sleep health. Educational intervention programs are needed to help parents understand that lifestyle behaviors may be related to sleep health.

Children with disabilities are at a higher risk of sleep problems than children without disabilities [9, 22]. Parents of children with disabilities often report that children have difficulty falling asleep, restless sleep, snoring, and nocturnal awakenings [23, 24]. In our study, most caregivers reported that their children had nightmares and nocturnal awakenings. It has been reported that nightmares and sleep disturbances are common among children with post-traumatic stress disorder or childhood trauma [25, 26]. It is vital that caregivers have an appropriate understanding of children’s disabilities and comorbidities and the impact of health conditions on children’s sleep. Sleep problems have been shown to be associated with deficits in child cognitive and behavioral functioning [27, 28].

Caregivers of children with disabilities may experience more stress and depression than caregivers of children without disabilities [14, 29, 30]. Children’s sleep disturbances may also adversely affect caregivers, such as mothers [9, 14, 22, 31]. Children with disabilities such as ADHD and cerebral palsy are more likely than children without disabilities to wake their parents at night [32]. Children’s sleep disturbances can be stressful for parents [14, 33]. Our study revealed that caregivers had adverse emotional responses such as anxiety and frustration because of children’s sleep problems. Maternal depression symptoms have been reported to adversely influence children’s sleep [34]. The provision of long-term care for children with disabilities may create great challenges for caregivers and impair their physical and psychological health [4]. Caregiving stress and other health issues from the challenges that caregivers of disabled children experience should be considered in designing effective family-based intervention programs that target children with disabilities, their caregivers, and family members.

There is much uncertainty about the responsibility that caregivers or health professionals should take for children’s sleep health. It may be important for both caregivers and health professionals to acknowledge their respective responsibilities for children’s sleep. Health professionals are well positioned to provide educational messages to children and caregivers. In this study, 8 out of 18 rehabilitation providers reported having professional experience with sleep medicine or sleep hygiene, whereas only 2 had sleep medicine or sleep hygiene training. Our study highlights the need for health professionals of children with disabilities to acquire necessary sleep medicine and sleep hygiene training to further assist children and their caregivers in promoting healthy sleep.

It has been reported that good sleep hygiene practices are associated with better sleep [35], while parent-set bedtimes are related to earlier bedtimes, longer sleep, and better daytime functioning [36]. In this study, we found that caregivers used various methods to address children’s sleep problems. Some caregivers used strategies such as story-telling, evening bathing, or providing comfort and affection to their children as a means for encouraging sleep. However, some caregivers relied on sleep medications or allowed children to watch television until they became bored and finally fell asleep. There are no ideal sleep medications for children; all hypnotic drugs tend to be effective for short periods, but may cause significant adverse effects [14]. Although the American Academy of Sleep Medicine has endorsed the use of melatonin for circadian rhythm sleep disorders [37], melatonin is beneficial only when melatonin secretion is inadequate or inappropriately timed [14, 38, 39]. Because sleep drugs should be prescribed only when appropriately implemented behavioral interventions are ineffective [14], caregivers should acknowledge the importance of promoting children’s sleep hygiene and sleep-related lifestyle behaviors such as physical activity and the control of electronics and stimulant use.

Public health recommendations are that children have an established bedtime routine and refrain from caffeine consumption and from sleeping in bedrooms with televisions [35]. The Institute of Medicine (IOM) has recommended that caregivers adopt practices that promote age-appropriate sleep durations, create environments that ensure restful sleep, such as no screen media in rooms where children sleep; encourage practices that promote child self-regulation of sleep [40]. Health and education professionals should be trained in how to counsel parents about their children’s age-appropriate sleep durations. It is important for caregivers and families to create a sleep-friendly routine and environment for children. Our findings about the difference between overall recognition of the importance of sleep and the ability of caregivers to articulate positive sleep management strategies underscore the need to implement effective interventions among caregivers to improve child sleep health.

Our study has limitations. We included only 27 focus group participants consisting of caregivers and rehabilitation providers at a rehabilitation center in Chile. The results based on the small sample size from one study site may not be generalizable to other populations. However, such focus group discussions can provide in-depth reflections that reveal common themes among caregivers and rehabilitation providers. In addition, we recruited participants who volunteered to participate in the study. The generalizability of our conclusions may be limited, since study volunteers may be enriched with a population of caregivers and rehabilitation providers who were more knowledgeable than nonparticipants. Despite this, our results suggest that the findings may be valuable for informing the development of intervention programs aimed at improving sleep health among children with disabilities. Further sleep studies in larger and culturally divergent populations are warranted to confirm our findings. Given that interventions can be delivered in various ways (e.g., online resources, face-to-face), future research should address the preferred ways caregivers want to receive information and preferred delivery methods by health professionals.

## Conclusions

This qualitative study showed that caregivers exhibited awareness of behavioral, environmental, and family factors related to children’s sleep hygiene. However, many caregivers were unaware of the influences of lifestyle factors such as use of electronics and consumption of caffeinated beverages on children’s sleep. Our findings concerning the difference between overall recognition of the importance of sleep and the ability of caregivers to articulate positive sleep management strategies underscore the need to implement effective interventions among caregivers to improve child sleep health. While both caregivers and rehabilitation providers recognized the importance of sleep for children’s health and functioning, they differed in their understanding of how sleep hygiene practices influence sleep. Rehabilitation providers recognized the negative influence of electronics on sleep and the positive influence of sleep routines. In contrast, caregivers reported use of television/movie watching and stimulants as coping strategies for managing children’s sleep problems. Health professionals are well positioned to provide educational messages to children and their caregivers, and further training may improve their ability to assist children with disabilities, and their families in achieving positive health goals. The knowledge gaps identified in this study can inform the design of educational messages and training programs that address caregivers’ and providers’ perceptions and practices surrounding child sleep.

## Abbreviations

ADHD: Attention-deficit and/or hyperactivity disorder
CPASS: The Chile Pediatric and Adult Sleep and Stress Study.
